# Onabotulinumtoxin A Treatment of Drooling in Children with Cerebral Palsy: A Prospective, Longitudinal Open-Label Study

**DOI:** 10.3390/toxins7072481

**Published:** 2015-06-30

**Authors:** Eigild Møller, Søren Anker Pedersen, Pablo Gustavo Vinicoff, Allan Bardow, Joan Lykkeaa, Pia Svendsen, Merete Bakke

**Affiliations:** 1 Department of Odontology, Faculty of Health and Medical Sciences, University of Copenhagen, Copenhagen DK-2200, Denmark; E-Mails: eigild.moller@gmail.com (E.M.); alba@sund.ku.dk (A.B.); jsly@sund.ku.dk (J.L.); 2 Department of Neurology, Bispebjerg University Hospital, Copenhagen DK-2400, Denmark; 3 Departments of Pediatrics and Radiology, Hvidovre University Hospital, Hvidovre DK-2650, Denmark; E-Mails: sap@dadlnet.dk (S.A.P.); pablo.gustavo.vinicoff@regionh.dk (P.G.V.); 4 Gerbrandskolen, Copenhagen Municipal Dental Service, Copenhagen DK-2300, Denmark; E-Mail: piasvendsen42@gmail.com

**Keywords:** drooling, botulinum toxin, cerebral paresis, salivary flow, children

## Abstract

The aim of this prospective open-label study was to treat disabling drooling in children with cerebral palsy (CP) with onabotulinumtoxin A (A/Ona, Botox^®^) into submandibular and parotid glands and find the lowest effective dosage and least invasive method. A/Ona was injected in 14 children, Mean age 9 years, SD 3 years, under ultrasonic guidance in six successive Series, with at least six months between injections. Doses and gland involvement increased from Series A to F (units (U) per submandibular/parotid gland: A, 10/0; B, 15/0; C, 20/0; D, 20/20; E, 30/20; and F, 30/30). The effect was assessed 2, 4, 8, 12, and 20 weeks after A/Ona (drooling problems (VAS), impact (0–7), treatment effect (0–5), unstimulated whole saliva (UWS) flow and composition)) and analyzed by two-way ANOVA. The effect was unchanged–moderate in A to moderate–marked in F. Changes in all parameters were significant in E and F, but with swallowing problems ≤5 weeks in 3 of 28 treatments. F had largest VAS and UWS reduction (64% and 49%). We recommend: Start with dose D A/Ona (both submandibular and parotid glands and a total of 80 U) and increase to E and eventually F (total 120 U) without sufficient response.

## 1. Introduction

Drooling, *i.e.*, unintentional loss of saliva from the mouth is uncommon after the age of four [1]. However, in children with CP who may have mental and physical disabilities and abnormalities in facial morphology, dental malocclusion and open mouth posture, drooling is present in 40% at the age of 7–14 years and considered severe in 15% [1,2,3]. Anterior drooling is typically present outside meals and is classified either as anterior drooling over the lip margin or posterior with coughing and aspiration. It is associated with a great inconvenience for the children and their family and may be considered as more or less socially unacceptable when saliva runs down over the chin and makes clothes wet. Drooling caused by increased secretion is defined as primary sialorrhea, which in children may be related to irritation of the oral mucosa, teething or side effects of pharmacologic treatment. Conversely, secondary sialorrhea is drooling associated with an increased amount of saliva in the mouth due to insufficient drainage. Drooling in children with CP is often secondary sialorrhea, *i.e.*, most likely caused by oral motor dysfunction [4]. The submandibular glands secrete the majority of UWS, whereas the parotid contributes equally during stimulation from mastication and taste. The salivary flow is assessed by draining, spitting, suction and swab methods in roughly equivalent values. The swab method is the least reliable but the best choice in patients with poor ability to cooperate [5]. In healthy children 6–11 years of age, unstimulated whole saliva (UWS) measured by the swab method is 0.63 mL/min (range 0.14–1.30 mL/min) [6].

Many types of interventions have been used in children to reduce their drooling. These include surgery, medicine, intraglandular injection with botulinum toxin (BoNT-A and BoNT-B), and physiotherapy, training to improve sensory function, behavioral therapy for better drooling control, oral appliances, and acupuncture. In a Cochrane review, there was insufficient evidence to recommend one intervention over others [7]. However, a later evidence-based review supported the use of BoNT for sialorrhea and concluded that secretion and drooling were reduced 3–9 months after injections into the salivary glands [8]. To avoid misplacement and for safety reasons, injections of BoNT should be performed with guidance from ultrasound instead of anatomical landmarks, and careful assessment of the children should be carried out prior to the treatment [2]. The mechanism of BoNT is a prolonged transient inhibition of the parasympathetic synaptic transmission without neurodegeneration and interference with the release of norepinephrine from sympathetic nerve endings [9,10,11,12]. In addition, it is associated with changes in the composition of saliva and the saliva increased thickness 6–8 weeks post-treatment.

Few studies have systematically examined the relationship between the dose of BoNT, the treated salivary glands and the effect on drooling. It is generally recommended to inject doses of 10–50 U A/Ona into each submandibular gland or each parotid gland alone or into all four salivary glands, but there is no definitive conclusion regarding the effect of this treatment [2,13]. Other studies have proposed doses between 5 and 75 U in each parotid with a recommended standard of 30–40 U, and between 5 and 30 U in each submandibular gland doses with a recommended standard of 30 U [14,15]. In children with CP, a total dose of 50 U A/Ona in parotid and submandibular glands decreased the flow rate significantly during chewing a piece of gauze after 4 and 12 weeks when compared with placebo [16]. A total dose of 3000 U Rimabotulinumtoxin B (B/Rima) corresponding to 60 A/Ona in parotid and submandibular glands reduced the frequency and severity of drooling in children with CP but thickened saliva, and 5000 U B/Rima (corresponding to 100 U A/Ona) caused viscous saliva, aspiration and dysphagia [17]. However, recommendable doses specifically for children with CP are not described. In addition, the technique and treated glands vary among studies, and systematic information are lacking on efficacy, safety and side effects in long-term treatment with in children with CP [13,18,19].

The purpose of this prospective open-label study was to find the lowest effective dose of A/Ona and least invasive method with the best balance between the effect on drooling and side effects in children with CP by testing various doses and gland combinations in repeated treatments.

## 2. Results and Discussion

### 2.1. Outcome Measures

Assessments were made at the hospital before treatment with A/Ona in each of the six successive Series with increasing total doses (from 20 U in the submandibular glands to 120 U A/Ona in the submandibular and parotid glands) and again after 2, 4, 8, 12 and 20 weeks. A 25% deviation in time was accepted for the post-treatment assessments. The following assessments were made in a questionnaire completed by the children’s parents/primary caregivers: severity of drooling problems (VAS; 0–24: No/insignificant, 25–50: Acceptable/manageable, >50: Unacceptable/severe), drooling impact (score on frequency/severity; 0–7: No drooling/dry to constant drooling/extremely wet), and treatment effect (scale; 0: Free of symptoms, 1: Marked improvement, 2: Moderate improvement, 3: Unchanged, 4: Moderate aggravation, 5: Marked aggravation) [20]. The number of bibs, *etc.* used in the daily routine was also assessed.

The investigator assessed if perioral dermatitis was present and asked about side effects. He also measured the UWS flow rate with a modified swab method as the average from two subsequent 2 min collection periods. In each period, two conventional dental cotton rolls (8 × 38 mm) were tied together with dental floss and placed on the tongue with the floss hanging outside the closed mouth to prevent aspiration. An initial plan to use four cotton rolls and 3 min collection periods had to be changed to obtain acceptance. Before and after each period, the chin and lips were dried with a napkin. Cotton rolls and napkins were weighed before and after collection, and the saliva flow rate was determined as the weight increment (1 g equals 1 mL of saliva) per min. If the flow rate was 0.20 mL/min or less, additional saliva was collected with a pipette. The method error and reliability of the collection method were evaluated in repeated measurements each consisting of two subsequent collection periods and sampled with 15 min intervals in the 14 children completing the study. The mean UWS value was 0.87 mL/min (SD 0.53), the method error 0.09 mL and test–retest repeatability *r* = 0.86. Later, the saliva from the cotton rolls was extracted by centrifugation (1500 g) and the concentrations of sodium and potassium and total protein were determined independently and blindly [10,12].

#### 2.1.1. Treatment Effect, Drooling and Salivary Flow

**Figure 1:**
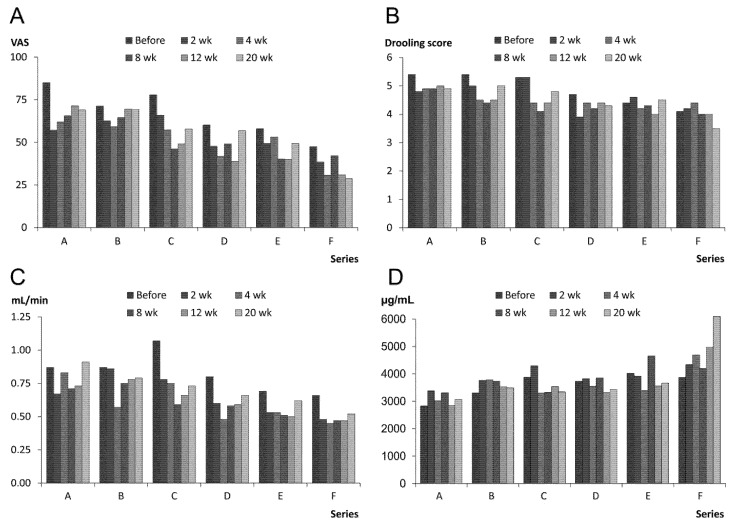
The columns represent the mean values of the outcome measures in 14 children with cerebral palsy (CP) treated with A/Ona (Botox^®^) for drooling (Series A–D with increasing total doses) before and 2, 4, 8, 12, 20 weeks (wk) after treatment, (**A**) visual analog scale (VAS; 1-100) for drooling problems; (**B**) drooling impact score (0-7); (**C**) saliva flow rate; and (**D**) total protein concentration.

The treatment effect reported by parents/primary caregiver was best in Series F and it increased from Series A to F (Table 1). Also the VAS for drooling problems in Series F was significantly lower than in Series A–E, and VAS in Series D and E was lower than in A and B (*p* ≤ 0.03; Figure 1A). The lowest VAS ratings were 2, 4, 8 and 12 weeks after treatment (greatest reduction 64% compared to pretreatment value; *p* ≤ 0.01). The drooling impact score in Series F was significantly lower than A–C, and D and E were also lower than A (*p* ≤ 0.01; Figure 1B). The greatest reduction of the drooling score compared to pretreatment value was at 8 weeks after treatment (28%; *p* ≤ 0.03).

The flow rate in Series F was significantly lower than in Series B and C, and Series E also differed significantly from A (*p* < 0.05; Figure 1C). The lowest flow rate appeared 2 and 4 weeks after treatment (maximum reduction 49% compared to pretreatment value (*p* < 0.05). Saliva total protein was significantly higher in Series F than in A–E, and the content in E was higher than in A (*p* < 0.05; Figure 1D), but there was no significant differences between weeks. The saliva sodium and potassium did not change significantly. The children did not develop dental caries lesions with the reduction of salivary flow rate.

#### 2.1.2. Prolonged Effect and Adverse Reactions

The baseline assessments obtained from the family/primary caregiver on VAS for drooling problems and score for drooling impact were significantly reduced (*p* < 0.05; Figure 1A,B) from Series A to Series E and F. In contrast to this, systematic reduction of baseline recordings of drooling from Series to Series with at least 6 months interval between injections there was no corresponding decline in the baselines of flow rates during the course of the study (*p* = 0.96; Figure 1C). Hence, a sort of prolonged effect was present as drooling VAS and score were less at the baseline of Series E and F than of Series A. These results together with the unchanged UWS may indicate improved oral clearance. In addition, there was a non-significant reduction in the average number of bibs, *etc.* from Series F compared to Series A (reduction 58%; *p* = 0.06). None of the children had persistent perioral dermatitis during the study period, but 11 had intermittent episodes, less frequent in Series F than in Series A.

Immediate and short-lasting side effects (Table 1) showed no direct and systematic relationship with dose or placement of A/Ona. Thus the side effects peaked with dose F given into both parotid and submandibular glands and also with treatment of the submandibular glands alone in series A. Also, the longer lasting eating and swallowing problems only occurred with the two highest doses (E and F). They were bothersome but did not require tube feeding.

**Table 1:** Reported treatment effect and side effects in 14 children with cerebral palsy (CP) completing six treatment Series with increasing doses of A/Ona (Botox^®)^ injected bilaterally into the submandibular glands alone (A: 10 U, B: 15 U, C: 20 U) or both submandibular and parotid glands (D: 20/20 U, E: 30/20 U, and F: 30/30 U).

Treatment Series	A	B	C	D	E	F
Treatment effect (0–5) ^a^ Mean (SD) of ratings (2, 4, 8, 12, 20 weeks) Median of ratings	2.6 (0.1) 3.0	22.5 (0.2) 2.5	2.3 (0.2) 2.3	1.9 (0.1) 2.0	1.9 (0.2) 2.0	1.5 (0.1) 1.0
Side effects (% of 14 treatments)	–	–	–	–	–	–
Immediate, short-lasting (less than 2 weeks after injection): Soreness, pain, swelling, dry mouth, viscous saliva	14.3	0.0	0.0	7.1	0.0	28.6
Long-term, longer lasting (up to 5 weeks after injection): Eating and swallowing problems (fluids and food)	0.0	0.0	0.0	0.0	14.3	7.1

^a^ Greater effect in Series F than in A, B and C, and greater effect in Series E and D than in A and B (*p* < 0.0001), *i.e.*, best effect with treatment of both submandibular and parotid glands (D–F), but also highest frequency of short-lasting side effects.

### 2.2. Discussion

Recent reviews on BoNT-treatment for drooling in children with CP and other neurological diseases, have pointed out problems concerning outcome measures, safety, dosage and choice of glands for injection [8,13,21]. We also worry about the long-term effects of recurring treatments, which may start when the child is five years old without knowing if or when they can stop. The present report deals with repeated BoNT treatment Series in 14 children over a period of 3–4 years. The study sample is relatively small, as the prevalence of CP is 2–3 children out of every 1000 and disabling drooling is only present in 40%. However, the systematic treatments with various doses and combinations of salivary glands together with the meticulous follow up allow for a realistic evaluation and recommendation regarding A/Ona treatment for drooling in children with CP. The reported treatment effect was best with treatment of both submandibular and parotid glands (D–F), and the flow rate was lowest in Series E and F which were also associated with the highest frequency of longer lasting side effects.

Internationally accepted reference values for UWS flow rates in healthy children do not exist, but it seems reasonable to characterize a flow over 1.30 mL/min measured with the swab method as highly increased [6,16]. Jongerius *et al*. [22] reported a submandibular flow rate of 0.40 mL/min (SD 0.19) in children with CP and a similar method error (0.11 mL/min) as in the present study. As 60%–70% of UWS is produced by the submandibular glands, the 0.40 mL/min corresponds to 0.57–0.67 mL/min UWS. Mean pretreatment UWS flow in the children completing the present study was slightly higher (0.87 mL/min). High secretion rates along with frequent dental malocclusion and insufficient lip closure indicate that the anterior drooling in the present study should be classified as a combination of primary and secondary sialorrhea.

The drooling problems were significantly reduced from pretreatment values to baseline values in E and F, and they stopped or were insignificant at the follow-up assessment in five of the children who completed the study. As the corresponding baseline flow rates were unchanged, this prolonged effect indicates that impeded and perhaps underdeveloped swallowing mechanisms may have improved or matured during the study period. A similar explanation could also apply to the stop of drooling in several children. As the drooling had stopped or was minimized in 41% of our entire cohort of 22 children, it indicates that the condition may improve spontaneously. This should be taken into account before surgical interventions for drooling in children or when evaluating various types of functional therapies. The spontaneous improvement may also explain part of the high dropout rate in our study in combination with the time and effort that were needed to participate.

A majority of trials used either 20–25 U A/Ona or equivalent BoNT doses in each submandibular or submandibular and parotid gland, corresponding to the doses in our Series C–F. In the analysis of outcome measures based on these doses, a time course with start of effect 1–2 weeks after BoNT-A, maximal effect at 8 weeks and return to base line after about 6 months were consistent findings. An effect still present after 1 year, most likely indicates stop of drooling [23]. With respect to UWS, previous studies have shown a reduction from 30% to 40% [10,24]. In the present study the UWS reduction was 49%, and since BoNT-A only blocks the parasympathetic innervation, this is probably the maximum obtainable reduction.

In previous studies, information from parents and caretakers on reduction on drooling scales ranged from 20% to 40%, fitting with the 28% reduction in the present study [24,25]. The reductions were 25% to 60% based on counts of saliva drops passing over the lips [25,26,27]. Our maximum reduction of 64% of VAS for drooling problems exceeds earlier findings, ranging from 30% to 40% [26,27]. Previous studies observed significant reductions of 20%–30% of the number of bibs, *etc.* used per day, while our reduction of 58% only appeared as a tendency.

Concerning saliva composition, we found a significant increase of total protein in Series E and F compared to A. A similar increase has been reported previously after treatment with BoNT-B [12]. The increase of total protein and the associated thickened saliva could explain both immediate and longer lasting side effects in Series E and F. Although saliva is important for clearance of the oral cavity and protection of soft and hard tissues, no side effects were reported with respect to oral health and decay of the dentition. We ascribe this to the repeated oral hygiene instructions.

In children of comparable age, short-lasting side effects such as soreness, pain, swelling, dry mouth, viscous saliva and dysphagia have previously been reported in 10%–45% of the treatment series with BoNT-A and in 30%–40% with BoNT-B [12,17,23,26,27]. The doses in these reports were similar to the present Series C–E with short-lasting side effects in only in 2% of the treatments. Longer lasting side effects such as eating and swallowing problems after treatment of submandibular and parotid glands were reported in 25%–35% of treatment series with BoNT-A and BoNT-B [17,28]. In the present study, with the comparable doses in Series E and F, longer lasting side effects were observed in only 11% of the treatment series. Longer lasting eating and swallowing problems seemed to occur at random but our high percentage of short-lasting side effects (14%) with the lowest dose in series A could be due to start difficulties. The frequency of 29% with short-lasting side effects in Series F may be in line with the findings of Basciani *et al.* [17], using BoNT-B in a dose corresponding to our Series E and F. The longer lasting side effects have been ascribed to the thickened saliva occurring at maximum effect of BoNT-A at about eight week after treatment [29]. However, in the present study, the longer lasting swallowing and eating difficulties started 1–2 weeks after injection and lasted another 3–4 weeks. This time course fits more with diffusion of the injected A/Ona to the mylohyoid or digastric muscles. In contrast Reid *et al.* found evidence of improvement with respect to eating and speech over four weeks [23].

## 3. Experimental Section

### 3.1. Design and Procedure

The doses of A/Ona (Botox^®^; Allergan, Irvine, CA, USA) and gland involvement increased from Series A to F with at least six months between injections over 3–4 years. The first three Series included, respectively, 10, 15 and 20 U A/Ona in each submandibular, and the last three Series, 20, 30, and 30 U in each submandibular combined with 20, 20, and 30 U in each parotid. The effect was evaluated in each Series at baseline (before treatment) and after 2, 4, 8, 12 and 20 weeks at the hospital.

With ultrasonographic guidance (GE Logiq 9, “thyroid” settings; 12 MHz linear transducer) and using a 25 G needle and a 1 mL syringe, 100 U of A/Ona was reconstituted in 1 mL saline (investigators SAP and PGV). Each dose was given in the middle of the gland to minimize diffusion into surrounding tissues. Anesthesia and immobility were obtained by inhalation of servoflurane or intravenous thiopental and oxygen mask.

The protocol was approved by the Danish Health and Medicines Authority and Regional Committee on Biomedical Research Ethics (26112-2672) and undertaken in accordance with the Declaration of Helsinki.

### 3.2. Participants

Twenty-two children 5–15 years old treated for CP at the Department of Pediatrics, Hvidovre Hospital, with substantial anterior drooling problems (>50 on a 100 mm visual analog scale (VAS) from 0 (no/insignificant drooling problems) to 100 (severe/unacceptable drooling problems) were invited to participate. All accepted after written informed consent obtained from their parents or legal guardian as well as assent from the children if possible. The median age of the 22 children was 10 years old (male:female ratio, 12:10). Their demographics and levels of CP severity are shown in Table 2: Gross Motor Function Classification System (GMFCS) median IV, *i.e.*, walking ability severely limited even with assistive device, uses wheelchairs most of the time and may propel their own power wheelchair, and may participate in standing transfers; Communication Function Classification System (CFCS) median 4, *i.e.*, the person does not consistently alternate sender and receiver roles; and Intellectual disability (ID), median 3, *i.e.*, moderate retardation.

**Table 2:** Pretreatment demographics and classifications of Gross Motor Function (GMCS) and Communication Function (CFCS) of 22 children (10 girls and 12 boys; median age 10 years) with cerebral palsy (CP). Patients no. 1–14 completed all six Series (A–F). No. 15–22 did not complete and were not included in the analysis of outcome measures (No. 21 deceased from CP-related disease and the rest were withdrawn from the study).

No.	Age (year)	Sex (F/M)	Feeding (Oral/Tube)	GMFCS level (I–V)	CFCS level (1–5)	ID—Intellectual disability (1–4)
1	12	M	Oral	II	2	1
2	9	F	Oral	V	5	3
3	15	F	Tube	V	5	4
4	8	F	Tube	V	5	4
5	11	M	Tube	V	4	3
6	5	M	Oral	IV	3	2
7	5	M	Oral	IV	2	1
8	10	M	Oral	IV	4	3
9	9	F	Oral	IV	5	4
10	12	F	Oral	IV	2	2
11	15	M	Tube	V	4	4
12	16	F	Tube	V	5	4
13	7	M	Oral	IV	3	2
14	5	M	Oral	IV	3	1
15	7	F	Oral	III	2	2
16	12	M	Oral	III	2	1
17	5	M	Oral	II	2	2
18	10	F	Oral	IV	4	3
19	10	M	Oral	IV	3	3
20	11	F	Tube	V	5	3
21	13	F	Tube	V	4	4
22	15	M	Oral	II	2	1
14 children completing all treatment series: Mean (SD)				IV (I)	4 (1)	3 (1)
8 children not completing all treatment series: Mean (SD)				IV (0)	3 (1)	2 (1)
All children: Mean (SD)				IV (I)	4 (1)	3 (1)

At admission, their height and weight were 108–165 cm (median 132 cm) and 15–50 kg (median 29 kg) and their BMI 11.83–21.40 (median 15.88). Seven of the children had percutaneous endoscopic gastrostomy (PEG) and were tube fed. No children had known history of aspiration. Sixteen were treated with various drugs. The most frequent were baclofen (*n* = 10), valproate (*n* = 6), oxcabazepine (*n* = 3) and levopromazepine (*n* = 3). Pharmacological treatment for drooling was discontinued several weeks before pretreatment examination to avoid confounding. Other medications were accepted. None of the children were in oral motor training programs for drooling. Four had increased secretion (>1.30 mL/min); most had periodic or chronic perioral dermatitis, insufficient lip closure, and more than half had distinct malocclusion (Table 3) [6].

**Table 3:** Pretreatment oral characteristics and drooling parameters in 22 children (10 girls and 12 boys; median age 10 year) with cerebral palsy (CP). Patients No. 1–14 completed all six Series (A–F). No. 15–22 did not complete and were not included in the analysis of outcome measures (No. 21 deceased from CP-related disease and the rest were withdrawn from the study).

No.	Insufficient lip closure (Y/N)	Distinct malocclusion (Y/N)	Perioral dermatitis (Y/N)	Drooling problems (VAS 0–100)	Drooling impact score (0–7)	Saliva flow rate UWS (mL/min)
1	N	N	N	100	5	0.90
2	Y	Y ^a,e,g^	N	82	5	0.12
3	Y	Y ^a,g^	Y	82	5	1.22
4	Y	Y ^b^	Y	96	6	0.42
5	Y	N	Y	80	6	1.25
6	Y	Y ^a^	Y	80	4	1.28
7	Y	Y ^a,d–g^	Y	72	6	0.50
8	N	N	N	68	4	0.45
9	Y	Y ^a,e^	Y	80	6	0.36
10	Y	N	Y	99	6	0.98
11	Y	Y ^b,c^	Y	85	4	0.38
12	Y	Y ^b,c^	Y	95	7	1.36 ^h^
13	Y	N	Y	95	6	0.92
14	Y	N	Y	76	5	2.05 ^h^
15	Y	N	Y	54	4	2.43 ^h^
16	Y	Y ^a,g^	Y	52	4	0.63
17	Y	Y ^a,e^	Y	51	6	0.73
18	N	N	N	63	6	0.63
19	Y	Y ^b,c,f^	Y	99	5	0.76
20	Y	N	Y	84	7	1.10
21	Y	Y ^a,d^	Y	84	6	1.09
22	Y	Y ^b,c^	Y	99	7	3.91 ^h^
14 children completing all treatment series: Mean (SD)				85 (10)	5 (1)	0.87 (0.53)
8 children not completing all treatment series: Mean (SD)				73 (19)	6 (1)	1.40 (1.10)
All children: Mean (SD)				81 (15)	6 (1)	1.10 (0.80)

^a^ Frontal open bite; ^b^ Extreme maxillary overjet; ^c^ Distal molar occlusion; ^d^ Mesial molar occlusion. ^e^ Posterior cross bite; ^f^ Deep bite; ^g^ Teeth crowding; ^h^ Increased secretion rate.

**Figure 2:**
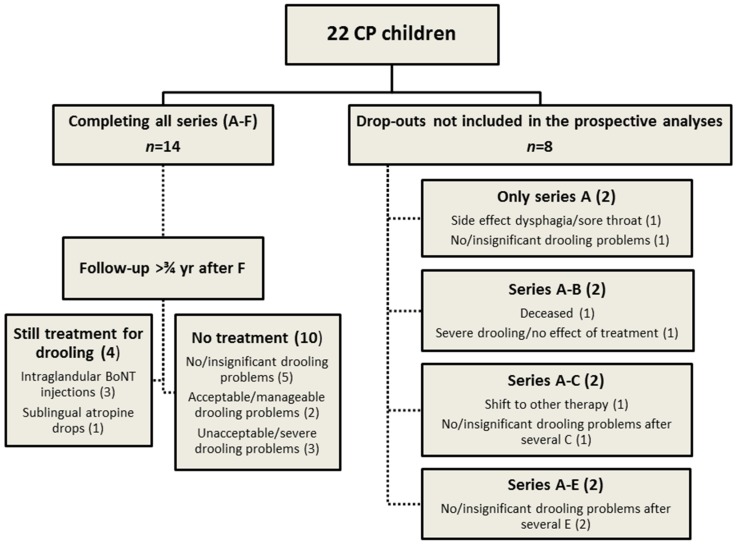
Flow chart describing the children with CP participating in the study and the dropouts: 14 children completed all six Series (A–F), and eight were withdrawn from the study by their parents/caregivers for various reasons and therefore not included in prospective analyses of the outcome measures. Severity of the drooling problems (100 mm VAS): No/insignificant, <25; Acceptable/manageable, 25–50; unacceptable/severe >50.

Before A/Ona treatment, they had no untreated dental caries. Supplementary to routine municipal dental service, they had extra oral examinations and oral hygiene instructions once per treatment series as reduced salivary flow is a risk factor for oral health and caries development (investigator PS). Fourteen of the 22 children completed the study and the six treatment series over 36–40 months. All children were offered to continue treatment after the study period, and at a follow-up 9 months after it ended, four children were still treated (three with BoNT injections and one with sublingual atropine drops) and 10 had no treatment (Figure 2). Seven children dropped out of the study for several reasons and one who died from CP-related disease. The group of children who completed the study and the dropouts did not differ significantly from each other (Table 2 and Table 3).

### 3.3. Statistical Analysis

The outcome data from each Series (A–F: Measurements before and after 2, 4, 8, 12, and 20 weeks after treatment) were evaluated with two-way analysis of variance (ANOVA) with *post-hoc* Tukey HSD test performed with respect to Series and week. ANOVA was also performed on the baseline values in each series. Comparisons of the characteristics between the group of children who did or did not complete the study and between the numbers of bibs in Series F *vs.* A were analyzed with *t*-tests. The statistical analyses were calculated using Statistica (StatSoft, Tulsa, OK, USA) and the level of statistical significance was set at *p* < 0.05.

Repeatability and method error of the assessment of the UWS flow rate were calculated by Pearson correlation analysis and Dahlberg’s formula (s(i)=√∑d^2^/2n; d is the difference between repeated measurements and n the number of children). Also, the frequency of perioral dermatitis and type of side effects were determined.

## 4. Conclusions

We recommend that the treatment of drooling with A/Ona should include injections in both submandibular and parotid glands guided with ultrasound, starting with 20 U in each gland and with insufficient response increase up to 30 U. The efficacy of A/Ona is reversible with a washout period of 20–30 weeks and consistent in repeated Series, providing a temporary relief for disabling drooling. Moderate side effects in terms of eating and swallowing problems lasting up to five week were present in 11% of the treatments with the two highest doses, and were probably due to diffusion of A/Ona. Since this is a small series and some children developed moderate dysphagia with higher doses, it is imperative to follow the swallowing function of the children (especially the younger ones) very closely after application of higher doses of A/Ona into parotid and submandibular glands.

As five of the 14 children completing all Series had no or insignificant drooling problems at the supplementary follow-up, CP patients must be followed closely to assess the need for further treatment and/or change of dose. Also, frequent oral hygiene instructions are recommended to prevent dental decay and preserve oral health when reducing the salivary flow rate.
